# Non-pharmacological interventions for sleep and quality of life: a randomized pilot study*

**DOI:** 10.1590/1518-8345.2598.3079

**Published:** 2018-11-14

**Authors:** Mariana Alvina dos Santos, Ana Paula da Conceição, Renata Eloah de Lucena Ferretti-Rebustini, Marcia Aparecida Ciol, Margareth McLean Heithkemper, Diná de Almeida Lopes Monteiro da Cruz

**Affiliations:** 1 Universidade Federal de Mato Grosso do Sul, Três Lagoas, MS, Brazil.; 2 Instituto Dante Pazzanese de Cardiologia, São Paulo, SP, Brazil.; 3 Universidade de São Paulo, Escola de Enfermagem, São Paulo, SP, Brazil.; 4 University of Washington, Department of Rehabilitation Medicine, Seattle, WA, United States of America.; 5 University of Washington, Department of Behavioral Nursing and Health Informatics, Seattle, WA, United States of America.

**Keywords:** Sleep, Sleep Hygiene, Phototherapy, Quality of Life, Heart Failure, Nursing

## Abstract

**Objective:**

to estimate the effects of non-pharmacological interventions to improve the quality of sleep and quality of life of patients with heart failure.

**Method:**

pilot study of a randomized controlled trial with 32 individuals assigned to four groups. Sleep was assessed using the Pittsburgh Sleep Quality Inventory, while health-related quality of life was assessed using the Minnesota Living with Heart Failure Questionnaire, at the baseline and at the 12^th^ and 24^th^ weeks. The means of the outcomes according to intervention groups were compared using analysis of covariance; effect sizes were calculated per group.

**Results:**

all groups experienced improved quality of sleep and health-related quality of life at the end of the intervention (week 12) and at follow-up (week 24), though differences were not statistically significant (p between 0.22 and 0.40). The effects of the interventions at the 12^th^ week ranged between -2.1 and -3.8 for the quality of sleep and between -0.8 and -1.7 for quality of life, with similar values at the 24^th^ week.

**Conclusion:**

the effects found in this study provide information for sample size calculations and statistical power for confirmatory studies. Brazilian Clinical Trials Registry - RBR 7jd2mm

## Introduction

Nurses have an essential role in the care provided to patients with heart failure (HF), teaching self-care and encouraging adherence to treatment^1^ ^-^ ^3^. Quality of life of these patients can be improved when clinical conditions are well-managed and under control. Nursing interventions are essential to relieving symptoms that limit wellbeing in persons with HF, especially among outpatients. 

Sleep changes negatively influence wellbeing and quality of life among populations with cardiovascular diseases^1^ ^-^ ^2^, impairing self-care practices^3^, and increasing the risk of unplanned hospitalizations^3^ ^-^ ^4^. Sleep disorders are associated with the level of severity of the disease, as its progression can cause difficulties to falling asleep and maintaining sleep, and negatively affecting the lives of patients with HF^5^ ^-^ ^7^.

Studies have reported an association between sleep and quality of life in various populations of patients^8^, including those with HF^9^, and it is believed that interventions aimed to decrease sleep disorders improve the quality of life of these patients^10^.

Various studies have used non-pharmacological interventions in different populations with the objective of improving quality of sleep. Interventions included cognitive-behavioral therapy (CBT)^11^ ^-^ ^12^, phototherapy^13^ ^-^ ^14^, the teaching of sleep hygiene habits^11^ ^,^ ^14^ ^-^ ^16^, and relaxation techniques^17^. Nursing interventions combining two or more therapies have also been described in the literature^18^ ^-^ ^20^, but little is known about the effects, mechanisms of action, and applicability of these interventions to improving the quality of life of individuals with HF.

Phototherapy refers to regular exposure to light and can be used to improve sleep. There is evidence that exposure to morning light benefits individuals with delayed sleep problems and/or seasonal sleep disorders^21^ ^-^ ^22^. One study conducted with institutionalized elderly individuals shows that light exposure during the morning improves total time of sleep during night^23^. Phototherapy is well-tolerated and presents very few adverse effects^22^. 

Sleep hygiene or sleep education is also a non-pharmacological treatment commonly used to improve sleep quality^24^ ^-^ ^27^. This practice consists of changing behaviors that hinder good quality sleep. Behaviors and habits that may harm sleep include: frequent day naps; intense physical activity at night; insufficient sun exposure; excessive consumption of caffeine and/or alcohol; smoking or eating in excess at night; excessive lighting and/or noise in the bedroom; and anxiety, among others^28^ ^-^ ^29^. One study compared two groups, where 17 patients were randomly assigned to the interventions and the group that performed sleep hygiene together with exercises presented improved sleep, while the control group presented no improvement^29^.

Resources destined to the health field are finite in any country; thus, low cost efficacious interventions are ideal. Therefore, there is a need to assess the feasibility and potential effect of non-pharmacological nursing interventions to improve the sleep patterns of individuals with HF.

In order to support the planning of a more controlled confirmatory study using a larger sample, a pilot study is usually conducted before a complex study with various interventions is implemented ^30^ ^-^ ^31^. This pilot study estimated the effects of non-pharmacological interventions on the quality of sleep and quality of life of patients with heart failure. 

## Method

This study followed recommendations provided by the Consolidated Standards of Reporting Trials (CONSORT)^32^. People with heart failure were recruited over a period of five months (July-November, 2013) from the HF outpatient clinic of a large cardiology service. The recruiting period defined the sample size for this pilot study of a randomized clinical trial. Inclusion criteria were: patients older than 18 years old with a medical diagnosis of HF; functional class I, II or III^33^; presenting stable clinical conditions that allowed them to participate in the study; and having telephone access. Exclusion criteria were: having cognitive impairment according to Folstein test^34^ or being a good sleeper (score≤5) based on the Pittsburgh Sleep Quality Index (PSQI)^35^, which is described in detail below.

Eligible individuals who consented to participate and signed free and informed consent forms were randomly assigned to one of the four intervention groups (eight individuals per group, totaling 32 participants): control; phototherapy; sleep hygiene measures; and a combination of phototherapy and sleep hygiene measures. The study was approved by the Institutional Review Board and, even though it is a pilot study, was registered in the Brazilian Clinical Trials Registry - RBR-7jd2mm.

The individuals were randomized in blocks using the Research Randomizer software (http://www.randomizer.org/form.htm) and each individual’s placement was put in opaque, sealed envelopes that were sequentially numbered. Immediately after randomization, demographic data and initial measures were collected from the participants, followed by the implementation of the intervention to which each participant was assigned. Initial orientation was provided by the researcher to all the participants individually in a private room and lasted 20 minutes. This orientation consisted of an oral presentation and a dialogue concerning the intervention and a leaflet containing the same information provided verbally was handed to the participants. All groups were followed-up via telephone with weekly calls during 12 weeks to reinforce the respective interventions and collect any information concerning potential intercurrences since the last contact.

The participants in the control group (C) received general guidance regarding their heart disease and the use of medications prescribed by their physicians, without specifically mentioning sleep problems.

The participants in the phototherapy group (PT) were instructed to have 40 minutes of sun exposure daily in the first half of the morning. The morning period was chosen to protect the patients against potential harm caused on the individuals’ skins by sun exposure.

The participants in the group addressing sleep hygiene measures (SHM) received instructions regarding habits that improve sleep, such as: not going to bed unless already sleepy; getting out of the bed if not asleep within 20 minutes; including relaxation activities in their daily routines before going to bed; keeping regular bedtime hours; not reading, writing, eating, watching TV, speaking on the phone, or playing cards in bed; not eating heavy meals (difficult to digest) close to bedtime; not exercising intensively within six hours before bedtime; not drinking coffee, black or mate tea, soft drinks, hot cocoa or alcohol from 4 to 6 hours before bedtime, and not smoking at least 4 to 6 hours before bedtime.

The participants in the group that received a combination of phototherapy and sleep hygiene measures (PT+SHM) received the instructions previously mentioned regarding both interventions. The participants in this group and those in the phototherapy group were instructed to apply sun protection before sun exposure. 

Neither the interventionist nor the participants were blind to the assigned group. To avoid biases in the assessment and interpretation of results, an evaluator who did not take part in the interventions collected data at the baseline and during follow-ups without knowing to which group the participants were assigned. The participants were instructed not to reveal the group to which they had been assigned during data collection. 

In weeks 4, 8 and 12, the participants were assessed in face-to-face visits. There was no contact with the participants between the 12^th^ and 24^th^ weeks, when they were assessed via telephone contact.

The initial assessment included demographic data (age, sex, marital status, occupation, and education) and clinical data (functional class measured by criteria of the New York Heart Association - NYHA^33^, type of medical follow-up, and the presence of dyspnea). Another four instruments were applied in the initial assessment to screen for symptoms. The Dutch Fatigue Scale (DUFS)^36^, adapted for Brazil^37^, measures the fatigue of individuals on a daily basis and its total score ranges from 8 to 40 points (the higher the score, the more intense the fatigue). The Dutch Exertion Fatigue Scale (DEFS)^36^, also adapted for Brazil, measures exertion fatigue and its total score ranges from 9 to 45 points (higher scores indicate more intense fatigue associated with physical effort). The Baecke Habitual Physical Activity Questionnaire, adapted for Brazil^38^, was used to measure the participants’ habitual physical activities for the last 12 months regarding three components: occupational activities, exercise, and leisure. The instrument is composed of 16 items assessing an individual’s pattern of physical activities over a long period of time in different contexts^38^ ^-^ ^40^. Depression symptoms were assessed using the Center for Epidemiological Studies - Depression (CES-D), adapted for Brazil^41^, with scores ranging from 0 to 60, (scores >15 indicate the presence of depressive symptoms).

The participants were assessed for primary and secondary outcomes at the baseline and after 4, 8, 12 and 24 weeks. The primary outcomes were quality of sleep and health-related quality of life. Adherence to the intervention was a secondary outcome. 

The Pittsburgh Sleep Quality Index (PSQI)^42^, adapted for Brazil^43^, was used to assess the subjective quality of sleep through seven sleep-related components. The sum of the scores obtained for each of the components results in a global score that ranges from 0 to 21 points (the higher the score, the worse one’s quality of sleep). The PSQI classifies individuals into good (≤5) and bad sleepers (>5)^43^.

The Minnesota Living with Heart Failure Questionnaire (MLHFQ)^44^ was adapted for Brazil and contains 21 items addressing the perception of patients regarding physical, socioeconomic and emotional aspects of HF. The MLHFQ measures health-related quality of life (HRQoL) and its total score ranges from 0 to 105 (scores <24 indicate good HRQoL, scores from 24 to 45 indicated moderate HRQoL, and scores >45 indicate poor HRQoL^45^. Change of 5 points are considered clinically significant^46^.

An Adherence to Intervention Index was developed by the authors to assess adherence to the intervention and was defined as the proportion of self-reported total number of days in which the intervention was actually implemented in relation to the total number of days in which the participant remained in the study.

The participants’ data were analyzed according to the groups in which they were assigned even when interventions were not followed as prescribed. Sociodemographic and clinical characteristics obtained at the baseline were compared between groups using Fisher’s exact text for categorical variables and the Kruskal-Wallis non-parametric test for the numerical variables. 

The means concerning the primary outcomes obtained in the 12^th^ and 24^th^ weeks were compared between the four groups through analysis of covariance, using the outcome’s initial value as the covariate^47^. The effect of each intervention (in the 12^th^ and 24^th^ weeks) was estimated as the difference between outcome values (final minus initial), divided by the initial outcome’s standard deviation^48^. The level of significance for all the tests was established at 0.05, without adjustment for multiple comparisons. These results should be interpreted with caution since this is a pilot study and the results will serve to support the design of future studies rather than be used as definitive confirmatory results.

The Kruskal-Wallis non-parametric test was used to compare the medians of percentage of adherence between groups and was chosen because it does not assume a specific distribution of the data. When the global test is statistically significant, *post hoc* pairwise comparison of the medians are performed using Dunn’s test ^(^ ^49^.

To explore the trajectory of the outcomes over the course of the follow-up weeks, a graph was created for each individual and group. Analyses were performed using the Statistical Package for the Social Sciences (SPSS) version 24 and R-Studio version 0.98.1074.

## Results

**Figure 1:**
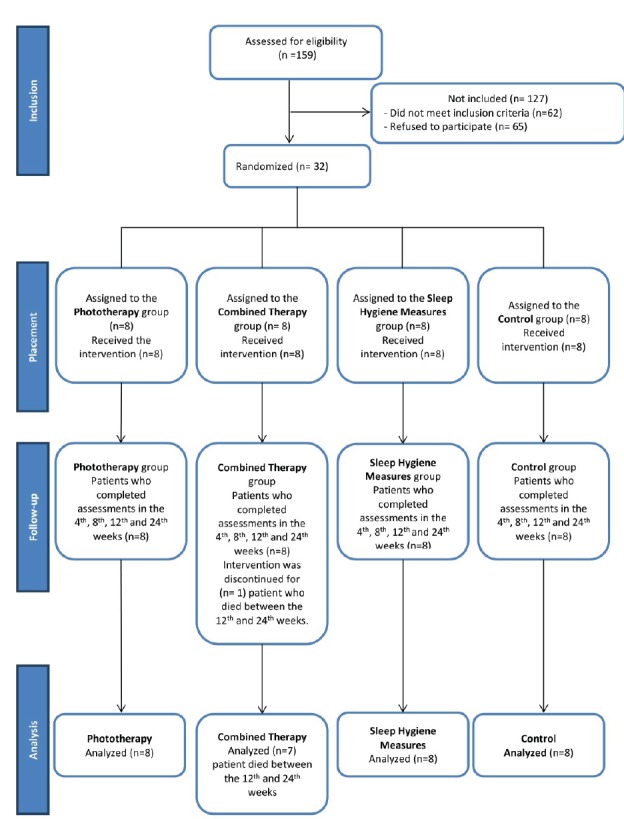
Follow-up flowchart. São Paulo, SP, Brazil, 2014

Of the 159 eligible patients, 62 were excluded due to cognitive impairment and 65 refused to participate (unavailable to attend follow-up visits, lived in another city, reported dermatological problems, or reported good quality of sleep, despite the assessment’s results showing they had poor quality of sleep). One participant assigned to the PT+SHM died between the 12^th^ and 24^th^ weeks. Figure 1 shows the follow-up flowchart.

Table 1 shows a summary of the participants’ sociodemographic and clinical characteristics at the baseline according to assigned group, showing the groups were comparable.

**Table 1:** Characteristics of the sample at the baseline according to the assigned group (N = 32). São Paulo, SP, Brazil, 2014

Characteristics	Control (n=8)	PT* (n=8)	SHM^†^ (n=8)	PT*+SHM^†^(n=8)	P-value^‡^
Males % (n)	50.0(4)	25.0 (2)	25.0 (2)	62.0 (5)	0.42
Age in years, Mean (SD^§^)	54.8 (6.9)	52.1 (11.8)	55.5 (12.4)	58.8 (11.0)	
Median (Min, Max)	55.5 (41-63)	51.5 (34-70)	57.5 (28-71)	58.0 (42-76)	0.49
Married, % (n) yes	88.0 (7)	88.0 (7)	62.0 (5)	75.0 (6)	0.79
Employment, % (n)					
Working (active)	12.0 (1)	25.0 (2)	12.0 (1)	-	0.69
Unemployed	12.0 (1)	12.5 (1)	12.0 (1)	25.0 (2)	
Retired	75.0 (6)	50.0 (4))	38.0 (3)	50.0 (4)	
On sick leave/ receiving financial support	-	12.0 (1)	38.0 (5)	25.0 (2)	
Schooling in years, Mean (SD^§^)	7.9 (6.1)	7.8 (3.0)	6.2 (3.4)	8.2 (5.9)	
Median (Min, Max)	5.5 (1-20)	7.5 (4-11)	7.5 (0-11)	9.0 (0-17)	0.89
Functional class - NYHA^║^, % (n)					
I	-	-	12.0 (1)	25.0 (2)	
II	62.0 (5)	62.0 (5)	38.0 (3)	25.0 (2)	0.57
III	38.0 (3)	38.0 (3)	50.0 (4)	50.0 (4)	
Follow-up, % (n)					
Medical consultation	3.0 (37.5)	5.0 (62.5)	3.0 (37.5)	5.0 (62.5)	0.65
Medical and nursing consultation	5.0 (62.5)	3.0 (37.5)	5.0 (62.5)	3.0 (37.5)	
Dyspnea % (n)	100 (8)	100 (8)	88.0 (7)	100 (8)	1
Cognitive state (MEEM^¶^), Mean (SD^§^)	28.0 (1.5)	27.5 (1.4)	26.8 (2.2)	26.4 (3.4)	
Median (Min- Max)	28.0 (25 - 30)	28.0 (26 - 30)	27.5 (22 - 29)	27.5 (21 - 30)	0.63
Fatigue (DUFS^**^), Mean (SD^§^)	29.2 (6.6)	29.4 (9.1)	28.4 (7.5)	26.4 (8.8)	
Median (Min - Max)	30.5 (17-37)	31.5 (15-39)	31.0 (13-36)	28.5 (12-36)	0.79
Exertion fatigue (DEFS^††^), Mean (SD^§^)	24.0 (8.8)	28.5 (14.0)	30.9 (14.1)	30.2 (14.7)	
Median (Min - Max)	25.0 (13-38)	27.0 (10-45)	34.0 (9-44)	34.5 (9-45)	0.72
Exercise (BHPAQ^‡‡^), Mean (SD^§^)	7.59 (1.39)	6.88 (0.96)	6.73 (1.09)	6.73 (1.79)	
Median (Min - Max)	7.56 (6.00-10.63)	6.81 (5.25-8.13)	6.56 (5.38-8.88)	6.62 (4.50-9.13)	0.50
Depressive Symptoms (CES-D^§§^), Mean (SD^§^)	22.4 (8.6)	19.3 (13.6)	28.2 (15.0)	18.1 (11.3)	
Median (Min - Max)	19.5 (12-36)	18.0 (6-50)	28.0 (9-52)	14.0 (6-37)	0.40

*FT - Phototherapy; †SHM - Sleep Hygiene Measures; ‡Kruskal-Wallis test for numerical variables or Fisher’s exact test for categorical variables; §SD - Standard Deviation; ║NYHA - New York Heart Association; ¶MEEM - Mini-Mental State Exam (scores from 0 to 30 points; the higher the score, the better one’s cognitive state); **DUFS - Dutch Fatigue Scale (scores from 8 to 40 points; the higher the score, the more intense one’s symptoms); ††DEFS - Dutch Exertion Fatigue Scale (scores from 9 to 45 point; the higher the score, the more intense one’s symptoms); ‡‡BHPAQ - Baecke Habitual Physical Activity Questionnaire; §§CES-D - Center for Epidemiological Studies - Depression (scores from 0 to 60, scores >15 indicate the presence of depressive symptoms)

Table 2 presents the means and standard deviations of the outcomes according to group and follow-up period. Even though the participants were randomly assigned to the groups, the PT+SHM group presented the lowest mean of initial PSQI (best quality of sleep) compared to other groups, a situation that may occur with very small samples. Over time, the means decreased in all the groups, and at the 12^th^-week follow-up, the SHM presented the lowest mean, followed by the PT+SHM, control and PT. The covariance analysis shows that the initial scores were important for the score obtained at the end of 12 weeks (p=0.02), but after adjustments, no difference was found between the groups (p=0.22). The lowest mean at the end of 24 weeks was obtained by the SHM, followed by the Control, PT+SHM and PT groups, with statistically significant initial scores (p=0.01) for the scores obtained at the 24^th^ week, while the differences between the groups were not significant (p=0.29).

**Table 2:** Means (standard deviation) of the primary and secondary outcomes according to follow-up and groups (N = 32). São Paulo, SP, Brazil, 2014

Outcomes	Week	Control	PT*	SHM^†^	PT*+ SHM^†^	p-value	
						Baseline	Group
Primary Outcomes							
Quality of sleep: Pittsburgh Sleep Quality Inventory - PSQI	0	12.4 (2.5)	12.4 (2.8)	12.0 (2.2)	10.5 (2.7)		
	4	5.8 (2.8)	8.9 (2.0)	5.8 (2.5)	6.0 (3.5)		
	8	4.2 (3.0)	7.4 (2.1)	4.5 (2.5)	6.1 (3.8)		
	12	5.6 (5.1)	7.4 (3.8)	3.6 (1.9)	4.4 (2.1)	0.02	0.22^‡^
	24	3.8 (2.8)	4.2 (1.8)	2.9 (0.8)	3.8 (1.6)	0.01	0.29^‡^
Quality of life: Minnesota Living with Heart Failure Questionnaire - MLHFQ	0	51.0 (19.1)	55.9 (24.3)	55.2 (25.3)	49.1 (28.3)		
	4	30.2 (17.6)	37.9 (21.8)	37.2 (22.8)	35.0 (21.4)		
	8	18.2 (9.3)	38.3 (21.7)	33.2 (21.9)	32.0 (26.4)		
	12	17.8 (14.7)	37.0 (19.9)	27.6 (28.4)	22.3 (22.1)	0.02	0.40^‡^
	24	14.9 (11.6)	30.6 (17.2)	20.2 (20.2)	21.8 (19.6)	0.02	0.35^‡^
Secondary Outcome							
% Adherence to the intervention	12						
Means (Standard Deviation)		94.2 (8.1)	74.0 (17.4)	90.9 (13.7)	78.0 (27.8)		
Median (Min.- Max.)		98.4 (78.0-100)	77.8 (41.0-95.0)	96.0 (60.0-100)	92.1 (30.0-98.0)		0.02^§^

*PT - Phototherapy; †SHM - Sleep Hygiene Measures; ‡Test of difference of means between groups using Covariance Analysis adjusted by initial values (week 0). §Kruskal-Wallis test for difference between groups of medians of intervention adherence. Missing data: one value was lost in the PT+SHM group at the 4th and 12th weeks and two were lost at the 24th week, one was lost in the PT group at the 8th week.

The means obtained by all the groups in the MLHFQ decreased (improved quality of life) over time. At the 12^th^ week, the lowest mean was obtained by the Control group, followed by the PT+SHM, SHM and PT. At the 24^th^-week follow-up, the lowest mean was obtained by the Control group, followed by the SHM, PT+SHM and PT. Again, the initial score obtained on the MLHFQ was important to explaining the outcome at the 12^th^ and 24^th^ weeks (p=0.02 in both follow-ups) but no statistically significant difference was found between groups (p=0.40 and p=0.35 for the 12^th^ and 24^th^ weeks).

Because this is a pilot study, the most important information refers to estimates of the effect of each type of intervention on the participants’ quality of sleep and health-related quality of life. Table 3 shows the estimates of the effects for the outcomes at the 12^th^ and 24^th^ weeks. The highest effect for the PSQI was found in the SHM group, followed by the Control and PT groups, with the PT+SHM group showing the lowest effect. The highest effect for the MLHFQ was experienced by the Control group, followed by the SHM group, with lower effects experienced by the PT+SHM.

**Table 3:** Estimates of the effects of interventions according to group and follow-up. São Paulo, SP, Brazil, 2014

Outcome	Interventions			
	Control	PT*	SHM^†^	PT*+SHM^†^
PSQI^‡^				
12 weeks	-2.7	-2.6	-3.8	-2.1
24 weeks	-3.4	-3.5	-4.2	-2.3
MLHFQ^§^				
12 weeks	-1.7	-0.8	-1.1	-0.9
24 weeks	-1.9	-1.0	-1.4	-1.1

*PT - Phototherapy; †SHM - Sleep Hygiene Measures; ‡PSQI Quality of sleep - Pittsburgh Sleep Quality Index, §MLHFQ Quality of Life - Minnesota Living with Heart Failure Questionnaire

**Figure 2:**
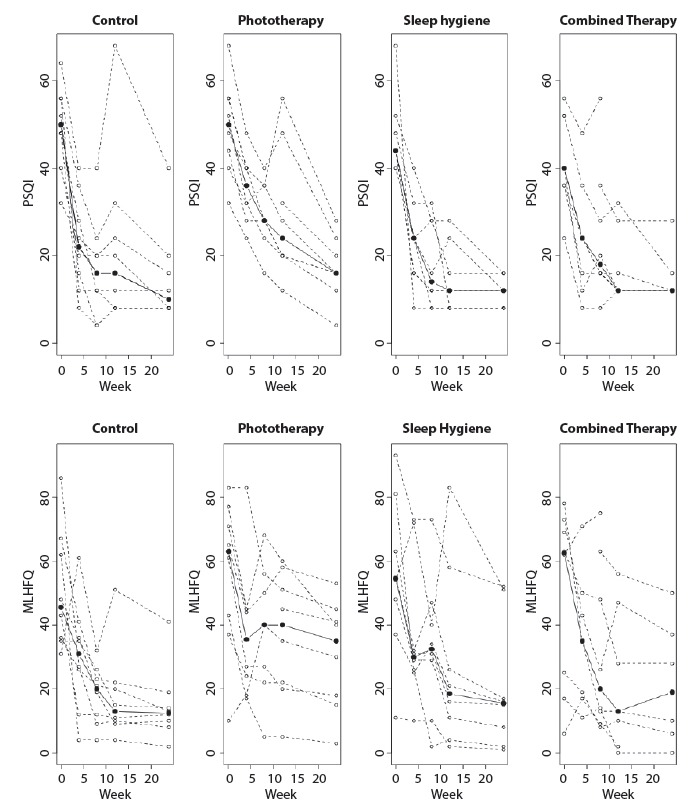
Trajectories of the scores obtained from the Pittsburgh Sleep Quality Index (PSQI) and in the Minnesota Living with Heart Failure Questionnaire (MLHFQ) according to follow-up and assigned group. São Paulo, SP, Brazil, 2014

Figure 2 shows the trajectory of outcomes for each individual per group and follow-up including during the intervention. The trajectory of the groups’ medians is represented by black dots.

The medians obtained in both the PSQI and the MLHFQ show that sleep patterns improved rapidly (a strong decline in week 4, followed by smaller declines) in all the groups, except for the PT group; the improvement obtained with the PT intervention was slower and did not reach the result obtained by the other groups.

Adherence was measured according to the percentage of days in which individuals followed the instructions according to the intervention to which they were assigned, in relation to the number of days in which individuals remained in the study. The results based on self-reports provided via telephone are presented in Table 2. Greater variation in terms of adherence to intervention was found in the PT and PT+SHM groups. According to the Kruskal-Wallis test, the medians of the percentages of adherence per group (98.4% Control, 96.0% SHM, 92.1% PT+SHM, and 77.8% PT) were statistically different (p = 0.02). *Post hoc* analysis showed that the PT group was statistically different from the Control group (p=0.04).

## Discussion

The four intervention groups presented improved quality of sleep and health-related quality of life at the end of the intervention period (12 weeks) and at the follow-up occurring in the 24^th^ week, though the differences found between the groups were not statistically significant. The main objective of this study, however, was to support future studies in this field of research and, for this reason, the discussion focuses on potential changes and suggestions for the design of studies based on the experience of this study.

According to the literature, individuals who practice SHM or PT tend to have better quality of sleep than those who do not implement any of these therapies^13^ ^-^ ^14^ ^,^ ^23^ ^-^ ^27^. Our conjecture was that a combination of SHM and PT would have a synergy and lead to improved results; however, this study did not obtain the expected results. The SHM group presented the best result, followed by the PT+SHM, PT and Control groups. The groups that included SHM may have obtained results in terms of quality of sleep because they have a well-defined component (instructions regarding sleep hygiene), which probably helped the individuals to adhere to the therapy. In this study, instructions concerning phototherapy included exposure to daily sunlight and a lack of effect may have been caused by poor adherence. Adherence may have depended on both the weather (having or not having sunlight) during the study’s period and on the individuals’ inability to expose themselves to available sunlight during the period necessary to obtain an effect. Initially, we expected that the Control group would not experience any improvement in sleep patterns. It is possible that the improvement experienced by the Control group is due to adherence to the self-care recommendations. Having knowledge of heart disease and correctly using medications may have an effect on an individual’s quality of sleep.

The same effects were observed for health-related quality of life among individuals with HF, with a decline of 33 points on the MLHFQ (5-point variations in the MLHFQ are considered clinically significant^46^). Among the four groups, the Control group obtained the best mean on the MLHFQ at the 12^th^ and 24^th^ weeks, showing that knowledge of the disease and encouragement to properly use prescribed medications may be a good intervention to improve patients’ quality of sleep and health-related quality of life. The Control group may not be considered a “pure” control because it was also contacted via telephone, a situation that does not normally occur in the care provided to these patients. The four groups received structured orientation and weekly telephone calls, intercalated by face-to-face visits; only the content of the orientation was different. The usual treatment provided in outpatient clinics does not include weekly telephone calls or reinforcement on how to manage symptoms, which can be considered a form of active intervention. If the purpose of future studies is to assess the effects of an intervention when compared to treatment that is usually provided, we suggest that the control group only receive treatment that is usually provided by outpatient clinics, without providing extra information or making extra contact. Otherwise, the effect experienced by the control group cannot be generalized to outpatients receiving the usual treatment. 

The use of control groups without treatment in clinical research is generally difficult to implement or is even unethical. The use of a control group receiving the usual treatment enables the assessment of an experimental intervention when compared to what patients usually receive over the course of their treatments, not affecting a study’s validity and utility^50^ ^-^ ^51^. In order to advance in knowledge concerning sleep patterns of individuals with HF, it is important to compare a group receiving usual outpatient treatment with a group receiving instructions regarding the management of symptoms and groups receiving PH and SHM. The possibility of being assigned to a control group may be a problem in recruiting participants, who may refuse to participate in the study if assigned to a control group or if they independently seek or receive information regarding the interventions provided to the other experimental groups^50^. One way to minimize the problem is to offer one of the experimental treatments to those originally assigned to a control group after the study is finished as a way to encourage participation.

Low cost, non-pharmacological therapies able to ease the management of the disease are desirable in any context of health. These therapies have the potential not only to improve quality of life, but also may lead to a decreased number of medical visits and unplanned hospitalizations. The interventions addressed in this study consisted of instructing individuals with HF at the beginning of the study and providing reinforcement and follow-up with patients via telephone calls. All the interventions were low cost, only involving the interventionist during face-to-face instructions and weekly telephone calls. 

All the interventions in this study improved the participants’ quality of sleep and health-related quality of life. Most individuals experienced improved sleep in the 4^th^ week of therapy in all the groups. As the duration of the participants’ diseases was different, it is unlikely that the results were only due to the natural progress of the disease. A common factor to all the interventions was the supply of self-care recommendations, focusing on the disease only (Control group) or on specific therapies to improve sleep, with reinforcement provided via telephone. Many studies have used telephone contact because it is a viable and low-cost alternative to implement interventions^52^ ^-^ ^56^, and it is also a way to improve adherence to non-drug treatment^55^. To continue this line of research, it would be important to compare these interventions with a pure control group (as mentioned before) and possibly to provide greater support that could promote self-care and sleep improvement within each therapy. It is very important to study the effects of these therapies on preventing intercurrences and unplanned medical visits and hospitalizations in long-term studies. 

In this study, self-reported adherence to therapy was quite high, though the phototherapy group reported a significantly lower adherence rate compared to the other groups. It is known that self-reported adherence tends to be overestimated compared to true adherence^57^, often motivated by the participant’s desire to please the researcher or clinical professional, a phenomenon that is called “social desirability”^58^. It is possible that the high level of adherence found in this study was due to this phenomenon. The participants in the phototherapy group, however, reported adherence rates 20% below that reported by the other groups and perhaps this difference is related to the therapy itself, that is, the need to have exposure to sunlight daily. Future studies should adopt different forms to assess adherence and ask the reasons for no or low adherence to the protocol.

The study was planned to provide an inexpensive and simple form of phototherapy. It is possible that participants forgot or missed the in the morning when they needed to get sun exposure, or that there were rainy or overcast days, or even that the instructions may not have been sufficiently motivating to encourage the participants to adhere to the protocol. In the future, we suggest that instructions include a motivational component (e.g., explain that it is possible to get sun exposure even on an overcast day). A more expensive solution would be the inclusion of phototherapy light bulbs, to which individuals would be exposed for a certain period each the day.

The high level of control over this pilot study, such as selecting collaborating researchers, preparing the nurse who performed the interventions, and ensuring that the same interventionist would address all the groups, was intended to ensure reliability in the study’s results. This study was conducted in a specialized university hospital and, for this reason, the sample may not represent the Brazilian population of individuals receiving treatment for HF as a whole. It was not possible to measure the intensity of sunlight to which the phototherapy group and the group receiving the combined therapies were exposed, which does not allow us to quantify the exact intensity of these groups’ sun exposure.

## Conclusion

This study showed the feasibility of the use of non-pharmacological therapies to improve quality of sleep and health-related quality of life among individuals with HF. Phototherapy and sleep hygiene measures, by themselves or in combination with other therapies, as well as knowledge regarding how to handle symptoms are low-cost interventions, both for patients and the health system, with the potential to promote sleep improvement. The next logical step in this line of research is to compare these interventions with a pure control group based on the usual treatment provided in the public service. Estimates obtained in this study can support sample size and statistical power calculations necessary for a confirmatory study.

Simple and low-cost therapies have the potential to improve the quality of sleep and quality of life of patients with HF, potentially influencing their health and the use of health services. Thus, these therapies should be investigated and eventually used in clinical practice. 
